# Research on the quantification and automatic classification method of Chinese cabbage plant type based on point cloud data and PointNet++

**DOI:** 10.3389/fpls.2024.1458962

**Published:** 2025-01-17

**Authors:** Chongchong Yang, Lei Sun, Jun Zhang, Xiaofei Fan, Dongfang Zhang, Tianyi Ren, Minggeng Liu, Zhiming Zhang, Wei Ma

**Affiliations:** ^1^ Country State Key Laboratory of North China Crop Improvement and Regulation, Hebei Agricultural University, Baoding, China; ^2^ College of Mechanical and Electrical Engineering, Hebei Agricultural University, Baoding, China; ^3^ College of Horticulture, Hebei Agricultural University, Baoding, China

**Keywords:** point cloud data, PointNet++, Chinese cabbage plant type classification, deep learning, clustering analysis

## Abstract

The accurate quantification of plant types can provide a scientific basis for crop variety improvement, whereas efficient automatic classification methods greatly enhance crop management and breeding efficiency. For leafy crops such as Chinese cabbage, differences in the plant type directly affect their growth and yield. However, in current agricultural production, the classification of Chinese cabbage plant types largely depends on manual observation and lacks scientific and unified standards. Therefore, it is crucial to develop a method that can quickly and accurately quantify and classify plant types. This study has proposed a method for the rapid and accurate quantification and classification of Chinese cabbage plant types based on point-cloud data processing and the deep learning algorithm PointNet++. First, we quantified the traits related to plant type based on the growth characteristics of Chinese cabbage. K-medoids clustering analysis was then used for the unsupervised classification of the data, and specific quantification of Chinese cabbage plant types was performed based on the classification results. Finally, we combined 1024 feature vectors with 10 custom dimensionless features and used the optimized PointNet++ model for supervised learning to achieve the automatic classification of Chinese cabbage plant types. The experimental results showed that this method had an accuracy of up to 92.4% in classifying the Chinese cabbage plant types, with an average recall of 92.5% and an average F1 score of 92.3%.

## 1 Introduction

In modern agricultural production, accurate quantification and classification of crop plant types are of great significance for variety improvement, crop management, and breeding processes (Donald, 1968; Mock and Pearce, 1975; Yuan, 2001). Particularly for leafy vegetables, such as Chinese cabbage, the differences in plant types are directly related to the growth conditions and yield (Sun et al., 2021). Traditionally, the classification of Chinese cabbage plant types has relied mainly on experienced agricultural workers who conducted manual observations, measurements, and subjective naming. This method is not only inefficient and lacks a unified scientific standard, but also fails to accurately distinguish certain plant traits that are difficult to describe verbally, such as leaf inclination and symmetry, leading to subjective and inconsistent results (Sun et al., 2014; Lai et al., 2019; Fan et al., 2021).

In recent years, with the rapid development of computer vision and machine learning technologies (Jordan and Mitchell, 2015; Yoo, 2015; Ayub Khan et al., 2021), image processing and deep learning methods for plant classification have gradually become popular research hotspot (Lee et al., 2017; Diaz et al., 2019; Kaya et al., 2019). S. Razavi et al. (Razavi and Yalcin, 2017) proposed a method using Convolutional Neural Networks (CNN) to classify plant types from image sequences collected from smart agricultural stations. This method helps to improve agricultural production processes, including pesticide application, fertilization, and timely harvesting, by automatically identifying different plant types. Sari et al. (Sari et al., 2020) used a Naive Bayes classifier and local binary pattern feature extraction in order to classify papaya types based on papaya leaf images. After preprocessing steps, such as grayscale conversion, image adjustment, and resizing, 150 papaya leaf images were used for training and testing. The results showed that the Naive Bayes classifier with specific pixel unit sizes and image adjustments achieved an accuracy of 96%. However, their research focused on 2D images, ignoring the three-dimensional structural information of the plants, which, to some extent, limits the classification accuracy and application range.

To overcome this limitation, researchers have begun to use point cloud data in order to capture and analyze the three-dimensional structure of plants in detail (Lou et al., 2015; Wang and Chen, 2020). Point cloud data comprehensively reflect the geometric morphology of plants from multiple perspectives, which is crucial for understanding plant growth conditions and classification. Currently, 3D point-cloud technology is a key tool in plant phenotypic analysis, particularly for characterizing the geometric and morphological features of plants. Li et al. (Li et al., 2020) proposed a new framework consisting of five stages for phenotypic analysis of the leaves of two ornamental plants, Caladium bicolor and Begonia masoniana. This framework includes multiview stereo point-cloud reconstruction, preprocessing, stem removal from the plant canopy, leaf segmentation, and leaf phenotypic feature extraction. Through experiments, the team calculated and compared phenotypic features such as single leaf area, length, width, and leaf inclination angle. However, after obtaining plant point-cloud information through 3D point clouds, the key challenge lies in accurately quantifying and finely classifying plants for better applications in practical plant phenotypic analysis and biological research.

With the rapid development of deep-learning technology, methods for processing point-cloud data are also evolving, particularly with the introduction of deep-learning models, such as PointNet and PointNet++ (Shrestha and Mahmood, 2019). These models can effectively handle complex 3D data from multiple perspectives, significantly improving the accuracy and efficiency of point-cloud data analysis by learning deep features within the data. However, the focus of current research combining plant 3D point-cloud data and deep learning is mainly on solving data segmentation problems. Ma et al. (Ma et al., 2024) in their study proposed a two-stage method that combined morphological features and deep learning point-cloud segmentation to extract banana pseudostem parameters. First, they used the DBSCAN clustering algorithm to extract seed points and completed the single-plant segmentation of bananas based on these seed points using a region-growing algorithm. They then applied PointNet++, PointNet, and a DGCNN to segment the pseudostem and canopy. This method effectively overcomes the challenge of single-plant segmentation in densely planted bananas and provides precise phenotypic parameter information for banana cultivation management. Applications of PointNet++ in plant species classification are relatively limited and mostly focused on tree classification. Liu et al. (Liu et al., 2022) used a backpack laser scanning (BLS) system to collect 3D point-cloud data for eight tree species from three regions. By designing comparative experiments, they explored the impact of different point cloud normalization methods on tree species classification accuracy and analyzed the effect of separating leaves and wood in the point cloud data on classification accuracy. Additionally, they tested five point-cloud downsampling methods to determine the most suitable downsampling method and demonstrated the potential of point-cloud deep learning methods in tree species classification.

In addition, recent advancements in computer vision for fruit detection and harvesting automation have provided new methods and insights for plant classification. For instance, Li et al. (Li et al., 2024) proposed a lightweight improved YOLOv5s model for detecting pitaya fruits in both daytime and nighttime light-supplement environments. The model not only enhances detection accuracy but also reduces computational resource requirements, making it suitable for real-time and resource-constrained environments. Chen et al. (Chen et al., 2024). investigated dynamic visual servo control methods for continuous operation of a fruit-harvesting robot in orchards. By dynamically adjusting the robot’s pose and position, these methods significantly improved the efficiency and reliability of fruit harvesting.

Building on this, our research further expands the application of deep learning classification models in agriculture. In this study, 257 samples of different varieties of Chinese cabbage were collected, and a multi-view image sequence method was used to quickly acquire and reconstruct the 3D structural information of the plants (Rong et al., 2021; Gao et al., 2022). We quantified the traits related to plant type based on the growth characteristics of Chinese cabbage. Subsequently, K-medoids clustering analysis was used for unsupervised classification of the data, and specific quantification of Chinese cabbage plant types was performed based on the classification results. Finally, we used an optimized PointNet++ model for supervised learning to automatically classify Chinese cabbage plant types.

## 2 Materials and methods

### 2.1 Data collection

The precision and resolution of the images play a crucial role in the subsequent 3D point-cloud construction and classification accuracy. High-precision images provide detailed and accurate information about the phenotypic traits of the Chinese cabbage plants, which is essential for capturing the fine geometric and color features needed for robust 3D modeling and classification. For image acquisition, we selected the FSFE-3200D-10GE camera from JAI in Copenhagen, Denmark. This camera is widely recognized in botanical research for its high resolution and high dynamic range, which effectively capture the detailed phenotypic traits of the plants. The high-precision camera ensures the accuracy of the obtained results, providing a solid foundation for the subsequent 3D point-cloud construction and classification tasks.

**Figure 1:**
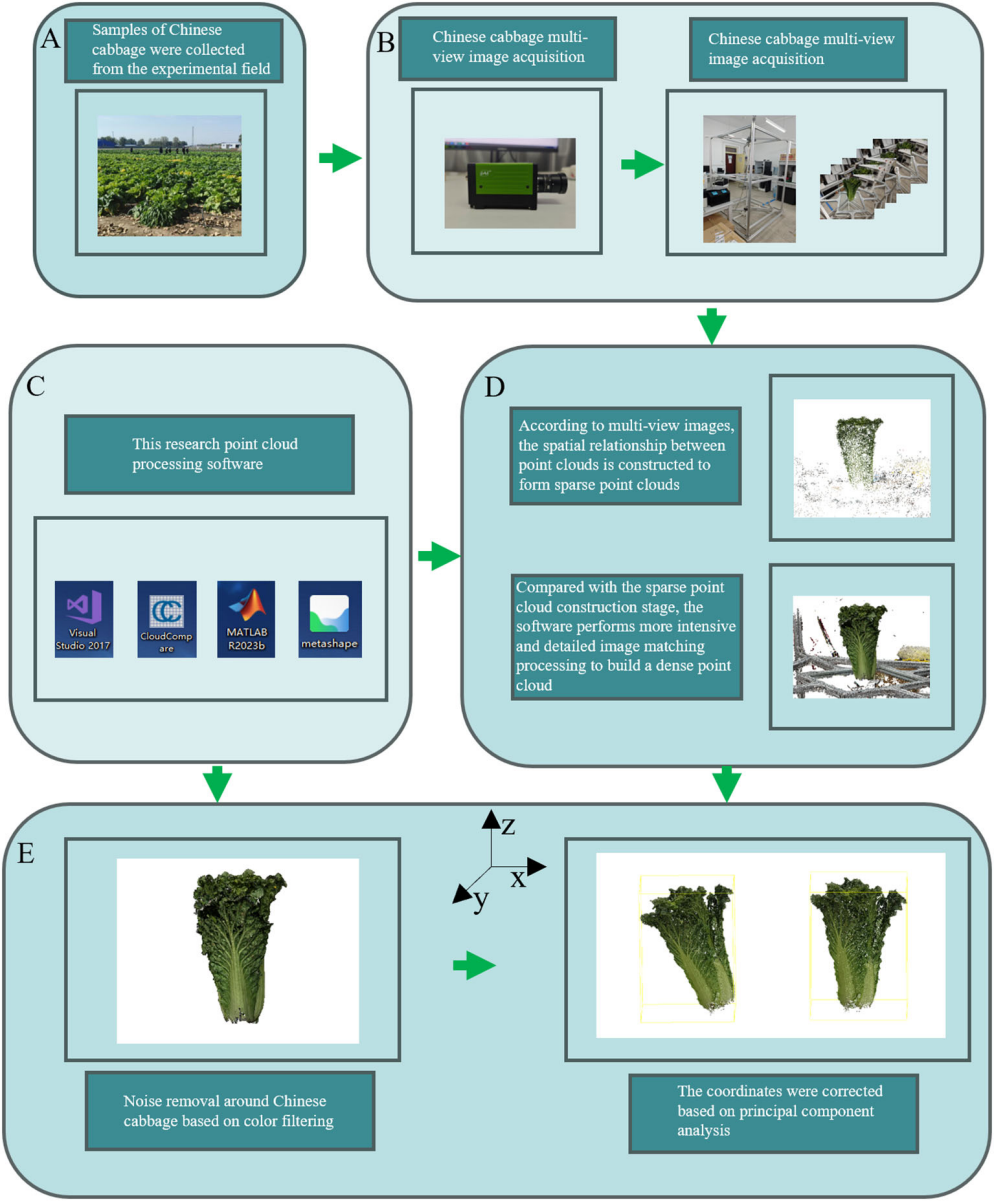
Workflow for Acquiring the 3D Point Cloud Models of Chinese Cabbage. The process begins with **(A)** Sample Collection, where Chinese cabbage samples are gathered from the experimental field. Next, in **(B)** Multi-View Image Acquisition, multiple images of the cabbage are captured using a multi-view imaging setup. This is followed by **(C)** Point Cloud Processing Software, where various software tools, including Visual Studio, CloudCompare, Matlab, and Metashape. In **(D)** Sparse Point Cloud Construction, sparse point clouds are generated based on the multi-view images, which are then refined into dense point clouds through detailed processing. Finally, **(E)** Noise Removal and Coordinate Correction involves removing noise using color filtering techniques and correcting the point cloud coordinates based on principal component analysis.

The complete process for obtaining 3D point-cloud data in this study is shown in **Figure 1**. The 257 Chinese cabbage samples analyzed in this study were carefully cultivated from multiple varieties with rich genetic backgrounds using *ex situ* conservation methods. All of the samples were planted in the experimental field of the Third Farm at the Western Campus of Hebei Agricultural University (latitude 115°25′ N, longitude 38°48′ E) and strict harvesting procedures were followed (**Figure 1A**). The image collection process was completed at the Artificial Intelligence Laboratory of Hebei Agricultural University (**Figure 1B**). Specifically, the image collection steps included placing the Chinese cabbage sample to be tested at the center of a platform with a transparent glass base (Zhang et al., 2022) and using a rotating camera to capture images around the sample, collecting 60 to 70 images per rotation.

### 2.2 3D point cloud reconstruction and preprocessing

After acquiring the multi-view image sequence, we used the Agisoft Metashape software for efficient 3D point cloud construction (Tinkham and Swayze, 2021). The Scale-Invariant Feature Transform (SIFT) algorithm was employed to identify key feature points in the images, and the Random Sample Consensus (RANSAC) algorithm was used to remove mismatches, thereby reconstructing sparse point clouds of the Chinese cabbage plants. We further applied clustering based on multi-view stereo (MVS) and a patch-based multi-view stereo algorithm to cluster, match, densify, and filter the point cloud, ultimately generating a high-density point cloud for the Chinese cabbage plants (**Figure 1D**).

Owing to the complexity of the environment and other uncontrollable factors, the reconstructed 3D point-cloud model inevitably contains noisy points. In order to improve the quality and accuracy of the point cloud, we adopted color-filtering techniques to remove these noisy points. The color-filtering method is mainly based on the significant color difference between the Chinese cabbage and the surrounding environment (Xiao et al., 2020). By comparing and analyzing the colors of the pixel points in the images with the preset color threshold of Chinese cabbage, we effectively identified and excluded noise points whose colors did not match the characteristics of Chinese cabbage (**Figure 1E**).

To ensure the handling of high-quality and uniformly standardized input data, we used Principal Component Analysis (PCA) to correct the coordinate axes of the point-cloud data, and we performed normalization to enhance the comparability and consistency between the different datasets. Through these steps, the data preprocessing workflow produced standardized high-quality 3D point cloud data, providing high-quality input for the automatic classification of Chinese cabbage plant types. This ensured the efficiency and accuracy of the classification system and provided reliable data support for the subsequent use of deep learning models for point-cloud feature learning and classification.

### 2.3 Extraction of phenotypic parameters of Chinese cabbage based on 3D point clouds

**Figure 2:**
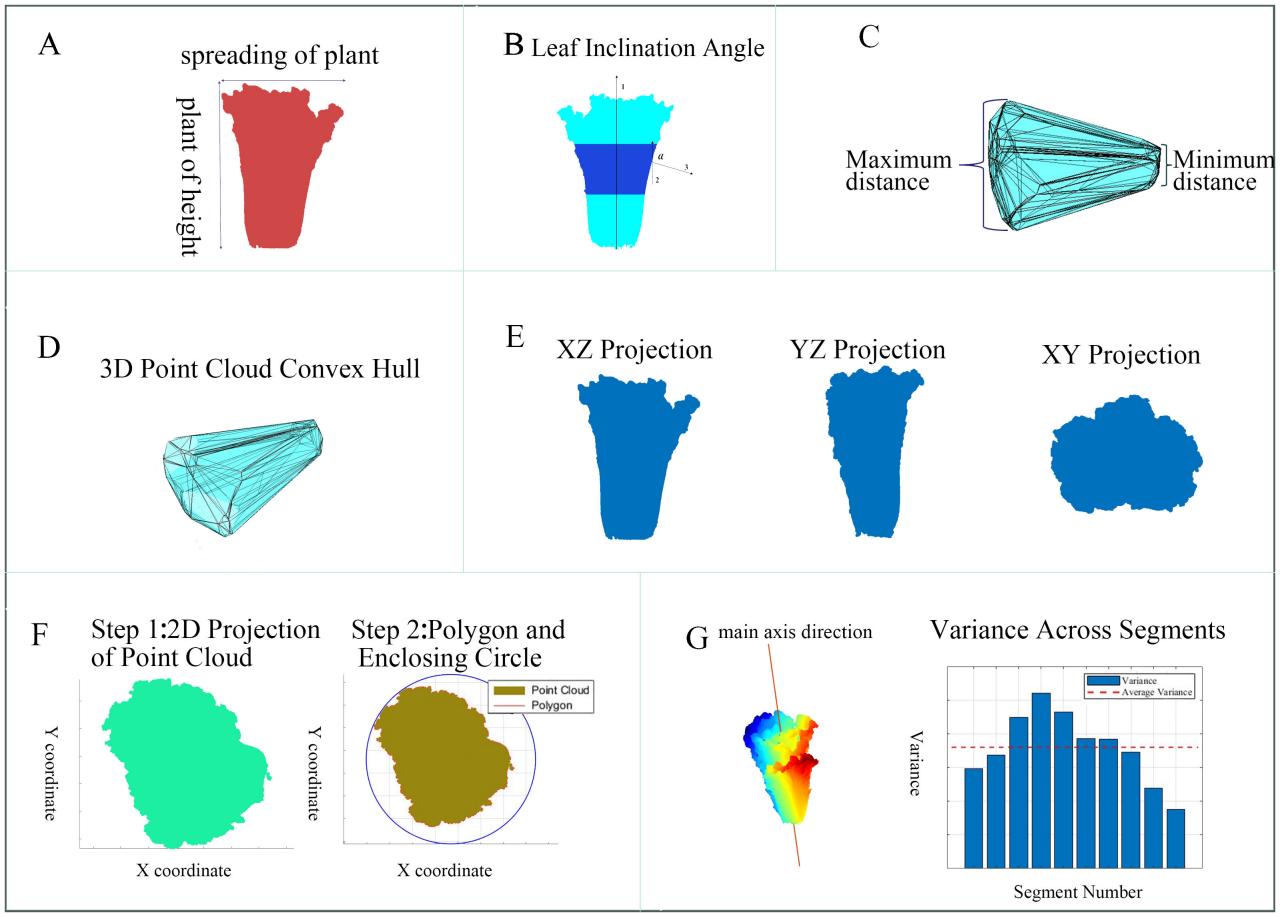
Extraction Diagrams of the Various Phenotypic Parameters of Chinese Cabbage. **(A)** Height-to-spread ratio, **(B)** Leaf inclination angle, **(C)** Radial dimension difference, **(D)** Volume and surface area, **(E)** Projection areas, **(F)** Head roundness, **(G)** Symmetry.

To comprehensively and accurately assess the differences between Chinese cabbage plant types and classify them effectively, we selected a set of parameters that included geometric, morphological, and structural features (**Figure 2**).

The selection of these parameters is based on several key reasons. First, the combination of geometric, morphological, and structural features can more comprehensively reflect the growth status and structural characteristics of Chinese cabbage, capturing subtle differences from different perspectives that a single feature type cannot fully assess. Second, dimensionless features, such as the plant height-to-plant spread ratio, leaf inclination angle, and radial dimension difference, are not affected by specific length units, providing better generalizability and comparability across different environments and scales, and avoiding errors caused by unit changes. Third, each parameter has a clear biophysical meaning, directly reflecting key growth characteristics of the plant. For example, the plant height-to-plant spread ratio evaluates growth patterns and balance, while the leaf inclination angle impacts the capture and utilization of photosynthetically active radiation, reflecting the plant’s health and potential yield. Finally, these features have high discriminative power in classification tasks, helping the model better identify and differentiate various types of Chinese cabbage. Volume and surface area provide overall geometric information, projection areas assess growth and expansion in different spatial directions, and head roundness and symmetry are important indicators for evaluating morphology and growth quality.

The software used to measure these parameters included CloudCompare, MATLAB 2023b, and Visual Studio 2017 configured with the PCL library. The basic calculation methods for the 10 phenotypic parameters were as follows:

Plant height-to-plant spread ratio ( T ): The ratio of plant height to plant spread is a fundamental parameter for evaluating the growth patterns of Chinese cabbage and provides an intuitive indicator of the balance between vertical and horizontal growth. This ratio was calculated by measuring the maximum height of the Chinese cabbage from the ground to the top (maximum Z-axis distance) and its diameter at the widest part of the horizontal plane (maximum XY plane distance) (**Figure 2A**).

Equation 1 expresses the process for calculating the plant height-to-plant spread ratio.


(1) $$T=\frac{H}{D}$$

where T is the plant height-to-plant spread ratio, H is the plant height, and D is the plant spread.

Leaf Inclination Angle ( $\bar{\theta }$ ): Leaf inclination angle is an indicator of the degree of tilt of the leaves relative to the vertical direction, directly affecting the capture and utilization of photosynthetically active radiation. The specific measurement steps were as follows. First, the Chinese cabbage was vertically divided into three equal parts, and the middle section was selected as the primary analysis object to exclude interference from the bottom and top parts. Then, the pcdenoise function was used to process the selected point cloud section to reduce the noise impact. The surface normals of each point in the point cloud were calculated using the pcnormal function. Next, the angle between these normals and the vertical direction (0, 0, and 1) was calculated. This angle is the deviation of the leaf from the vertical direction and reflects the leaf inclination angle (**Figure 2B**). As the leaves face different directions, the final angle obtained is the absolute value of the angle between the leaf normals and the vertical direction. Finally, the leaf inclination angles of all the measurement points were averaged in order to obtain the average leaf inclination angle of the entire Chinese cabbage plant.

Equations 2 and 3 express the calculation process for the leaf inclination angle.


(2) $$\theta_{i}=\cos^{-1}(|n_{i}\cdot z|)$$

(3) $$\bar{\theta }=\frac{1}{N}\sum_{i=1}^{N}\theta_{i}$$

where $n_{i}$ represents the normal vector of each point; z is the vertical direction vector; $\theta_{i}$ is the leaf inclination angle of each point; N is the number of points; and $\bar{\theta }$ is the average leaf inclination angle.

Radial Dimension Difference ( W ): Measuring the radial dimension difference of the Chinese cabbage in the XY plane can better assess the uniformity and consistency of its longitudinal morphology. The specific steps were as follows: The convhull function was used to find the outer contour of the point cloud on the XY plane, as the convex hull simplifies the point set, making subsequent calculations consider only the points forming the convex outer contour. Then, the distances between the contour points were compared to find the maximum and minimum distances and their differences were calculated (**Figure 2C**).

Equation 4 expresses the calculation process for the radial dimension difference.


(4) $$W=\max(d_{ij})-\min(d_{ij})$$

where W denotes the radial dimension difference, $d_{ij}$ the maximum distance between any two points in the XY plane, and $\max(d_{ij})$ the minimum distance.

Volume ( V ) and Surface Area ( S ): Measuring the volume and surface area can provide specific geometric information on Chinese cabbage. A point-cloud convex hull refers to the smallest convex polyhedron that closes point-cloud data. This convex polyhedron covers all data points. The overall volume and surface area of the Chinese cabbage were calculated (**Figure 2D**).

Equations 5 and 6 express the calculation processes for the volume and surface area, respectively:


(5) $$V=\frac{1}{6}\sum_{k=1}^{n}\left|\left(r_{i}\cdot (r_{j}\times r_{k})\right)\right|$$

(6) $$S=\frac{1}{2}\sum_{k=1}^{n}\left|r_{i}\times r_{j}\right|$$

where V is the volume; S is the surface area; $r_{i},r_{j},r_{k}$ is the vertex vector coordinate of the convex hull; and n is the number of vertices and faces of the convex hull.

Projection Areas ( S1 , S2 , and S3 ):The projection areas of the Chinese cabbages on the XZ, YZ, and XY planes were calculated to reflect their growth and expansion in different spatial directions (**Figure 2E**). These projection areas were obtained by projecting the 3D point-cloud data after principal component analysis onto the corresponding plane and then calculating the area of the convex hull covering the region.

Equations 7–9 express the calculation process for the projection areas.


(7) $$S1=\text{Area}(\text{convhull}((x_{i},z_{i})|\forall i)$$

(8) $$S2=\text{Area}(\text{convhull}((y_{i},z_{i})|\forall i)$$

(9) $$S3=\text{Area}(\text{convhull}((x_{i},y_{i})|\forall i)$$

where S1, S2, S3 is the projection area in the XZ, YZ, and XY planes. $x_{i}$ , $y_{i}$ , and $z_{i}$ represent the coordinates of the points in the three-dimensional point cloud data.

Head Roundness( O ): Head roundness is an important criterion for evaluating Chinese cabbage quality. The measurement of roundness is an essential assessment of morphology and growth quality, reflecting how close it is to a perfect circle. First, the point cloud of the Chinese cabbage was projected onto the XY-plane to extract its 2D contour information. Subsequently, the area of the polygon formed by these 2D points and the area of the minimum enclosing circle that can contain this area were calculated. The roundness of the Chinese cabbage was quantified by the ratio of the polygon area to the circular area (**Figure 2F**).

Equation 10 expresses the calculation process for head roundness.


(10) $$O=\frac{A}{A_{c}}$$

where O is the head roundness, a dimensionless ratio; the closer to 1, the more the shape approximates a perfect circle. A is the area of the polygon formed by the projection of Chinese cabbage on the XY plane. $A_{c}$ is the area of the minimum enclosing circle.

Symmetry ( M ): Measuring the symmetry of Chinese cabbages aims to quantitatively assess morphological regularity and balanced development. The specific steps are as follows: principal component analysis (PCA) was used to find the main axis direction of the point cloud and the point cloud of the Chinese cabbage was divided along the XY plane into several parts. The variance in distances from each point to the main axis for each part was calculated. Finally, we calculated the average of the variances for all parts. The smaller the mean and standard deviation of the distance differences, the better is the symmetry of the Chinese cabbage. This method allowed for the objective quantification of the morphological symmetry of Chinese cabbage, thereby assessing its growth status and quality (**Figure 2G**).

Equations 11 and 12 express the symmetry calculation process.


(11) $$\sigma_{k}^{2}=\frac{1}{S_{k}}\sum_{p\in S_{k}}(d(p,L)-\mu_{k}{)}^{2}$$

(12) $$M=\frac{1}{K}\sum_{k=1}^{K}\sigma_{k}^{2}$$

where $\sigma_{k}^{2}$ is the average variance, $\mu_{k}$ represents the mean distance of all points in the kth group to the main-axis L , $S_{k}$ represents the number of points in the kth group (i.e., the group size), d(p,L) represents the distance from point p to the main-axis L , M is the symmetry, and K is the number of groups.

### 2.4 Quantification and classification of Chinese cabbage plant types based on cluster analysis

In this study, the K-medoids clustering analysis method was used to perform a detailed structural exploration and group division of the dataset (Arora et al., 2016). Compared to other clustering algorithms, K-medoids provide more stable and reliable clustering results. The workflow of the cluster analysis begins with the z-score standardization of all phenotypic parameter datasets involved to eliminate differences in scales and ensure that each dimension has equal influence in subsequent analyses. After standardization, the number of clusters (k) was determined, which is a crucial parameter in cluster analysis that affects the granularity and distinguishability of the clustering results.

Next, the K-medoids algorithm randomly selects (k) data points as the initial cluster centers in the standardized dataset. For each point in the dataset, the algorithm calculates its distance to these (k) center points and assigns each point to the cluster represented by the nearest cluster center. This step ensures that the data points are grouped into clusters that are more similar (i.e., at the shortest distance).

Then, the algorithm enters the iterative process, recalculating the “center point” (i.e., “medoid,” the point in the cluster with the smallest average distance to other points) of each cluster, and then it reassigns each data point to the cluster represented by the nearest new center point. The iteration stops when the cluster composition no longer changes or reaches a predetermined number of iterations. This process ultimately determines the optimal clustering structure of the dataset. Each point is assigned to one cluster, with each cluster represented by a cluster center (medoid). The clustering results reveal the intrinsic organization and patterns within the dataset, providing a foundation for in-depth data analysis and understanding.

Through this method, the different groups present in the dataset could be clearly distinguished, allowing for an in-depth analysis of each group’s characteristics and their internal similarities and differences. This supports subsequent data application and research decisions.

### 2.5 Classification framework of Chinese cabbage plant types based on PointNet++

PointNet is a deep learning architecture for processing point-cloud data (Qi et al., 2017). Its core concept is to transform the input features and compare the input with the transformed data for tasks such as point-cloud classification, segmentation, and semantic annotation. However, the main limitations of PointNet are its weak perception of the local structure of point-cloud data and its lack of robustness to spatial transformations.

PointNet++ improves upon PointNet by introducing a hierarchical neighborhood structure for local feature learning and multiscale sensitivity analysis, thus enhancing the perception of the local point cloud data structure and robustness to transformations, aiming for more accurate point cloud processing in complex scenarios (Qi et al., 2017). Due to the structural complexity of Chinese cabbage plant types, PointNet may fail to capture sufficient local features, leading to decreased classification accuracy. However, the excellent local sensitivity and transformation robustness of PointNet + + can more accurately capture the characteristics of Chinese cabbage plant types, thereby improving the classification accuracy. Therefore, we chose to use PointNet++ to classify the Chinese cabbage point cloud plant types.

PointNet++ divides the point-cloud data of the Chinese cabbage into multiple overlapping local regions. In this process, it first captures fine geometric structures within small neighboring regions. These local features are then further organized into larger units and processed to produce higher-level features. This process was repeated until the feature representation of the entire set of points was obtained.

In the classification process of this study, we divided the classification into two main parts: feature extraction and fully connected layer classification. In the feature extraction stage, we processed the Chinese cabbage point cloud data using several set abstraction layers. Each abstraction layer involves sampling (selecting representative points), grouping (grouping points according to the point cloud density), and applying PointNet layers (extracting the features of each group). After these steps were completed, a feature vector containing 1024 elements was obtained.

**Figure 3:**
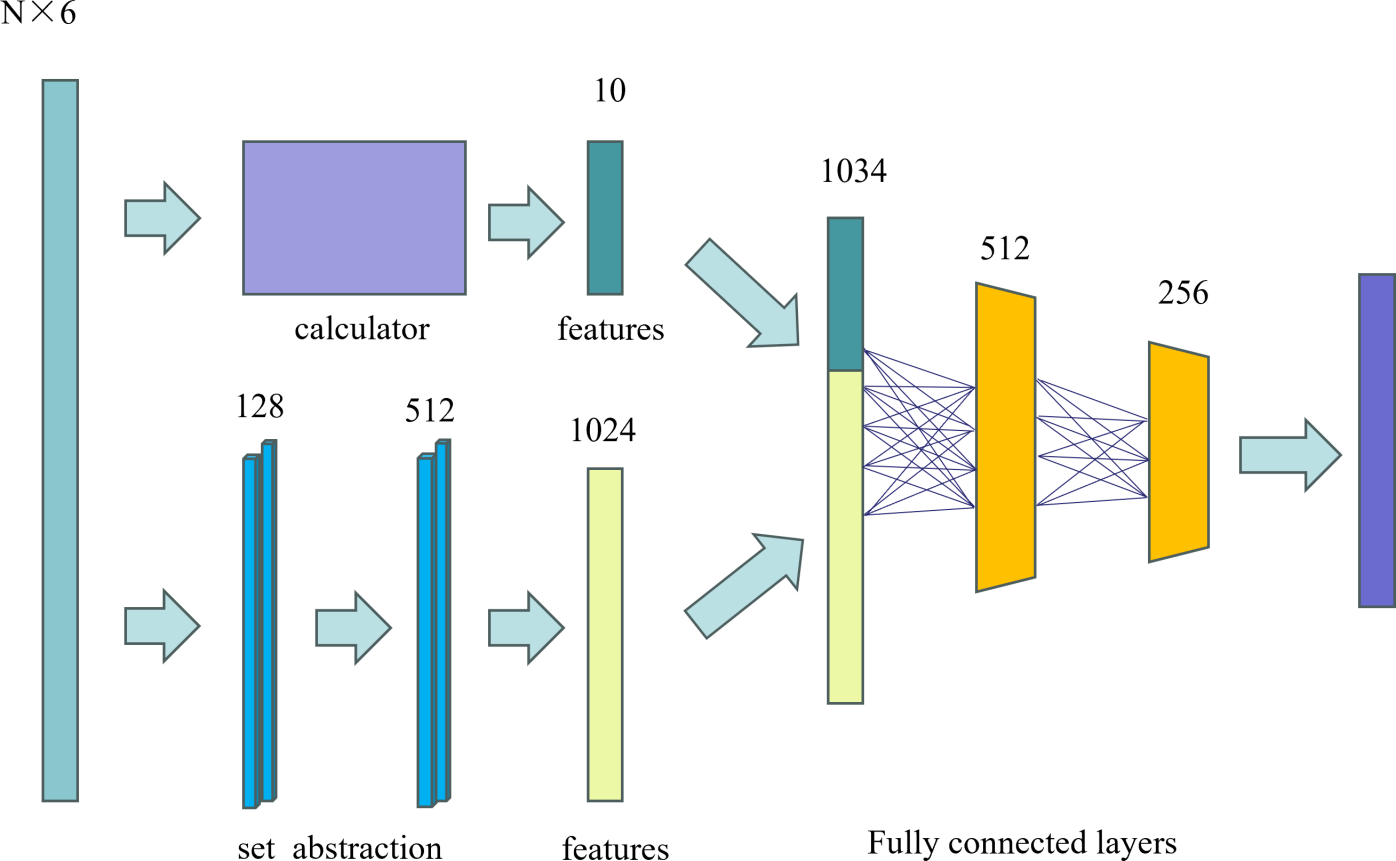
Network Structure for Chinese Cabbage Classification.

Additionally, we incorporated ten custom dimensionless features (the ten plant type parameters measured earlier) to capture the specific geometric and biological characteristics of the Chinese cabbage plant type (Zhou et al., 2022). These features were then combined with the 1024-dimensional feature vector to form a feature vector with 1034 dimensions. Finally, this 1034-dimensional feature vector was classified through a fully connected layer. The structure of the Chinese-cabbage classification network is shown in **Figure 3**.

Owing to the varying number of points in the point clouds of each Chinese cabbage, downsampling was necessary to standardize the size of the dataset. This study used Farthest Point Sampling (FPS) as the downsampling strategy (Okada et al., 2023). For each Chinese cabbage point cloud dataset, the FPS was performed first. By iteratively selecting the farthest points, the distribution and coverage range of the sampling were ensured, thereby maintaining the characteristics of the original point cloud as much as possible during the downsampling process. Subsequently, a limit was imposed on the number of points in each point cloud to ensure that each point cloud contained 20,000 points. This process successfully addressed the issue of an imbalance in the number of points in the different point clouds in the dataset, thereby providing high-quality input data for subsequent deep learning model training.

The experimental dataset was randomly divided into two parts: 80% of the data was selected as the training set for model training and tuning, and the remaining 20% was designated as the test set to validate the model’s performance on unseen data. In addition, to increase the diversity of the test data and test the robustness of the model, the test set data were augmented by expanding the 205 samples in the test set to 900. This augmentation includes operations such as rotation, scaling, and addition of random noise to simulate potential real-world data variations. Each class in the test set was expanded to 225 samples in order to avoid a significant imbalance in the amount of data among the different classes in the training set. Ultimately, 952 samples were used for the model training and evaluation.

The deep-learning framework used in the experiment was PyTorch (2.0.0 + CUDA 11.8). The operating system used for the experiment was Windows 10, with the following computer configuration: 13th Gen Intel^®^ Core™ i5-13490F 2.50 GHz, 16 GB RAM, and NVIDIA GeForce RTX 4060Ti. **Table 1** lists the model hyperparameters and optimized configuration parameters used in this experiment, where multiple schemes were selected for experimentation. We will conduct hyperparameter tuning based on the hyperparameters and configuration parameters listed in **Table 1**. Through this process, we aim to analyze the impact of each hyperparameter on model performance and identify the optimal configuration combination.

**Table 1:** Configuration of the model hyperparameters.

Hyperparameter	Value	Declaration
Model	SSG/MSG	Two different point cloud sampling strategies
Batch size	4/8/12/16/20	The number of samples used for each training
Number of points Epochs	1024/2048/4096/8192 50/100/200/300/500	The number of points selected in each point cloud The number of complete training cycles on the entire data set
Optimizer	Adam/SGD	Method for updating network weights
Learning rate	0.001/0.01/0.1	Control the number of steps the model takes to update parameters at each iteration
Decay rate Dropout Rate	0.0001/0.001 0.3/0.5	Weight attenuation to prevent overfitting Regularization techniques to prevent overfitting

## 3 Results

### 3.1 Clustering and quantification results of Chinese cabbage plant types

**Figure 4:**
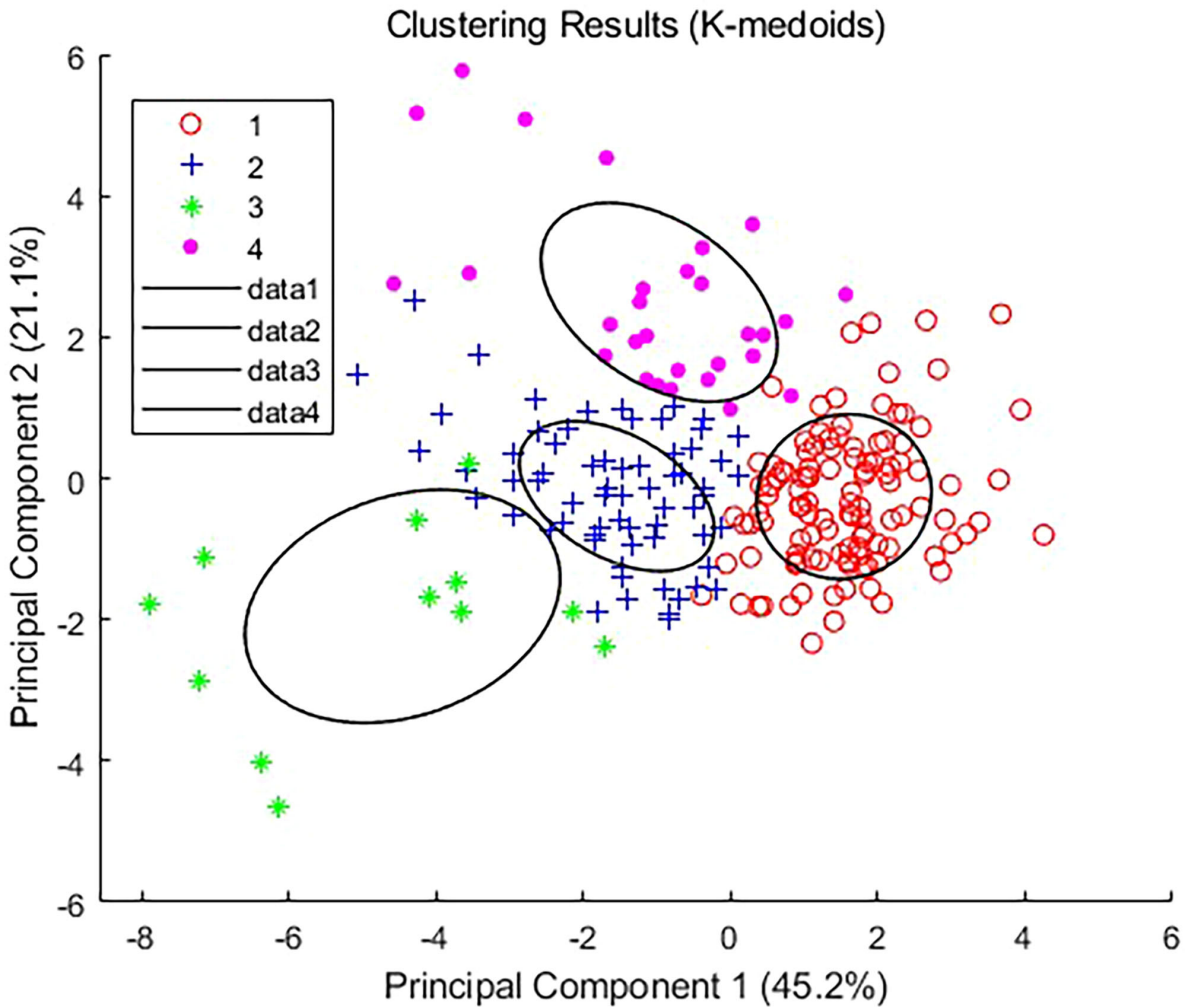
Classification of the Chinese Cabbage Plant Types Based on K-medoids.

**Figure 5:**
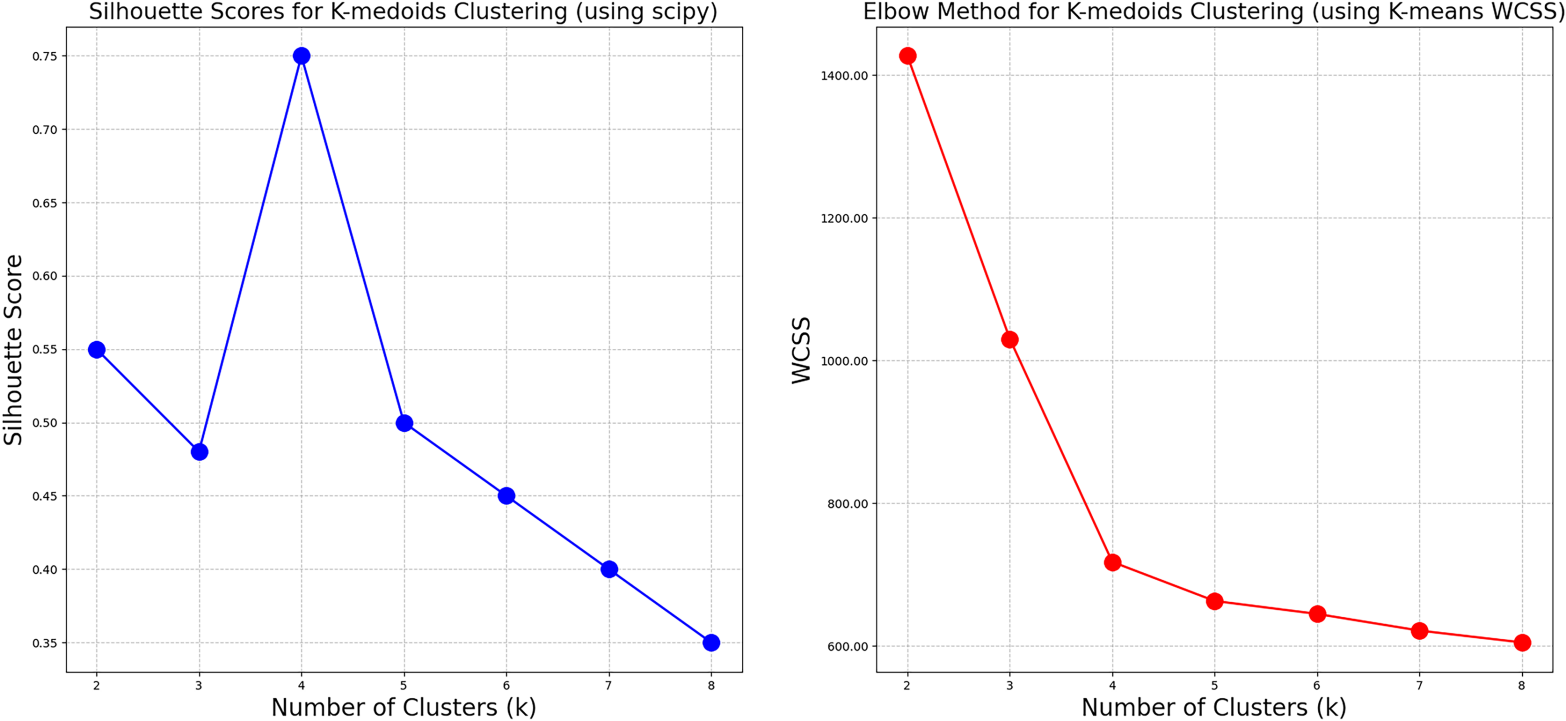
Comparison of contour coefficient and WCSS with the number of clusters.

After conducting a K-medoids cluster analysis (**Figure 4**), the Chinese cabbage plant types were categorized into four main clusters. This choice is based on the following considerations (**Figure 5**): We tested the silhouette scores for different numbers of clusters (k=2, 3, 4, 5, 6, 7, 8). The silhouette score, ranging from -1 to 1, indicates better clustering as it approaches 1. In our analysis, the silhouette score reached its highest value at k=4, suggesting that the internal structure of the four clusters is more compact and the separation between different clusters is greater, thus achieving the best clustering effect. Additionally, we used the K-means algorithm to calculate the WCSS (Within-Cluster Sum of Squares) for different k values. The WCSS decreases gradually as k increases, but there is a clear elbow point at k=4, further confirming that four clusters are optimal.

Based on the characteristics of each cluster (**Table 2**), four types of Chinese cabbage were identified: full type (Cluster 1), highly lean type (Cluster 2), tight type (Cluster 3), and round spread type (Cluster 4). The ratio of plant height to plant spread was low, with a large projection area, volume, and surface area on the x-z and y-z planes, resulting in a relatively full body size at all angles. The tall and lean type exhibited well-balanced parameters, with a large ratio of plant height to plant spread and a symmetrical tall and lean shape. The compact type had a small projected area, a thin appearance from all angles, and a small blade inclination and volume. Finally, the circular spread type featured wide-spreading plants on the side with a large xy-plane projection area and surface area, along with high roundness.

**Table 2:** Analysis of the phenotypic differences between the Chinese cabbage groups.

	T	S1	S2	S3	R	V	S	O	M	W
CLUSTER 1	1.1274	0.8359	0.7769	0.7046	60.9285	0.2484	2.1247	86.6433	0.1862	0.9157
CLUSTER 2	1.5136	0.4127	0.4109	0.4555	67.8658	0.1457	1.5216	105.3768	0.1638	0.6823
CLUSTER 3	1.2058	0.2217	0.1768	0.3443	61.4423	0.0422	0.5850	155.3771	0.0877	0.4127
CLUSTER 4	1.4600	0.7689	0.632	1.6705	70.3238	0.1739	1.699	372.2589	0.1764	0.7195

**Figure 6:**
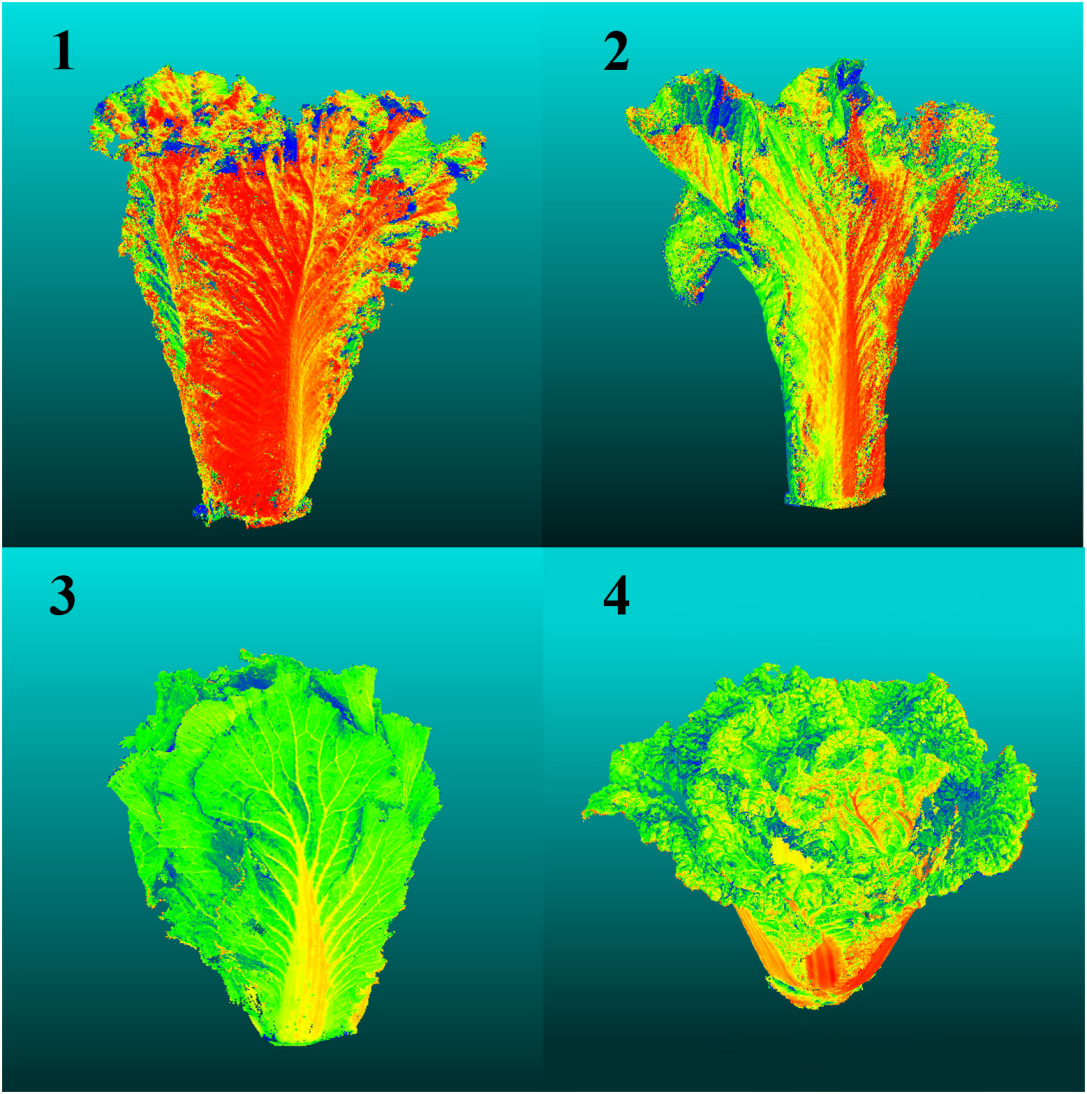
Schematic Diagrams of the Four Plant Types.

A diagram depicting the four plant types is shown in **Figure 6**.

To validate the effectiveness and efficiency of the automatic classification method, we designed a control group experiment involving five experienced agricultural experts who manually screened the samples into four major categories of Chinese cabbage. The results showed that the time required for manual screening was significantly higher than that for the automated method, and the accuracy was also notably lower. Specifically, the experts had to distinguish each sample individually, with an average classification time of 10 seconds per sample for each expert. In contrast, the automated method could classify all samples within a few seconds.

**Figure 7:**
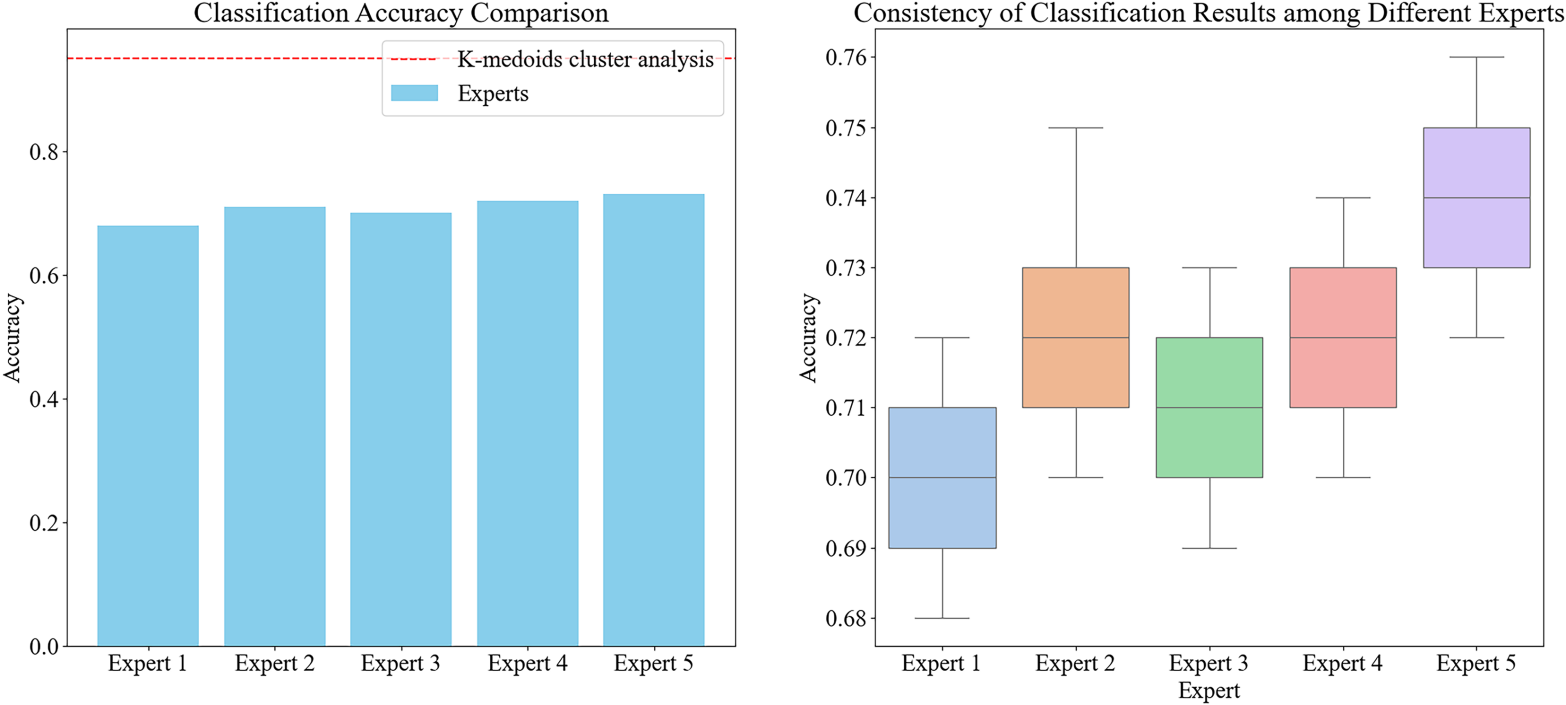
The accuracy of classification for different experts and the consistency of classification results for five experts, noted: In the graph on the left, the bar chart represents the average classification accuracy of each expert, and the red dashed line represents the classification accuracy of K-medoids cluster analysis. In the figure on the right, the box plot shows the distribution of the classification results of the five experts, showing the degree of consistency and variation among different experts.

Moreover, even when accounting for the time required for the initial point cloud data collection and measurement, the total time consumed by the automated method was still higher than that of manual screening. However, manual screening was heavily influenced by subjectivity and the fatigue of the human eye, resulting in large differences in classification results between different experts and even within the same expert each time (**Figure 7**). This resulted in low consistency and accuracy. The automated method, through a unified algorithm and standard, ensured the consistency and reproducibility of the classification results, making it a more reliable and efficient choice overall

### 3.2 Model training results

Through the iterative experiments and cross validation, we fine-tuned the parameters of our model to achieve optimal performance. We employed the Multi-Scale Grouping (MSG) strategy for model selection. The MSG strategy groups and extracts features from point-c ata at multiple scales, capturing richer local and global information, which enhanced the model’s ability to identify complex Chinese cabbage plant types.

We chose a batch size of eight in order to balance computational resource utilization and accelerate the training process while ensuring stable gradient estimates. The number of points was set to 4096 to ensure efficient computation and adequate capture of the 3D morphological features of the cabbages for classification. Training was conducted over 200 epochs, providing ample time for the model to learn and optimize, resulting in stable performance.

We used the Adam optimizer, which is known for its fast convergence and stable performance, making it suitable for complex deep learning tasks such as Chinese cabbage plant type classification. The learning rate was set to 0.001, allowing smooth parameter updates in each iteration, thereby avoiding instability and ensuring gradual optimization. We set the decay rate to 0.0001, which prevented overfitting and promoted gradual model optimization. Finally, we included a dropout rate of 0.5, which effectively prevented overfitting and enhanced the generalizability of the model.

**Figure 8:**
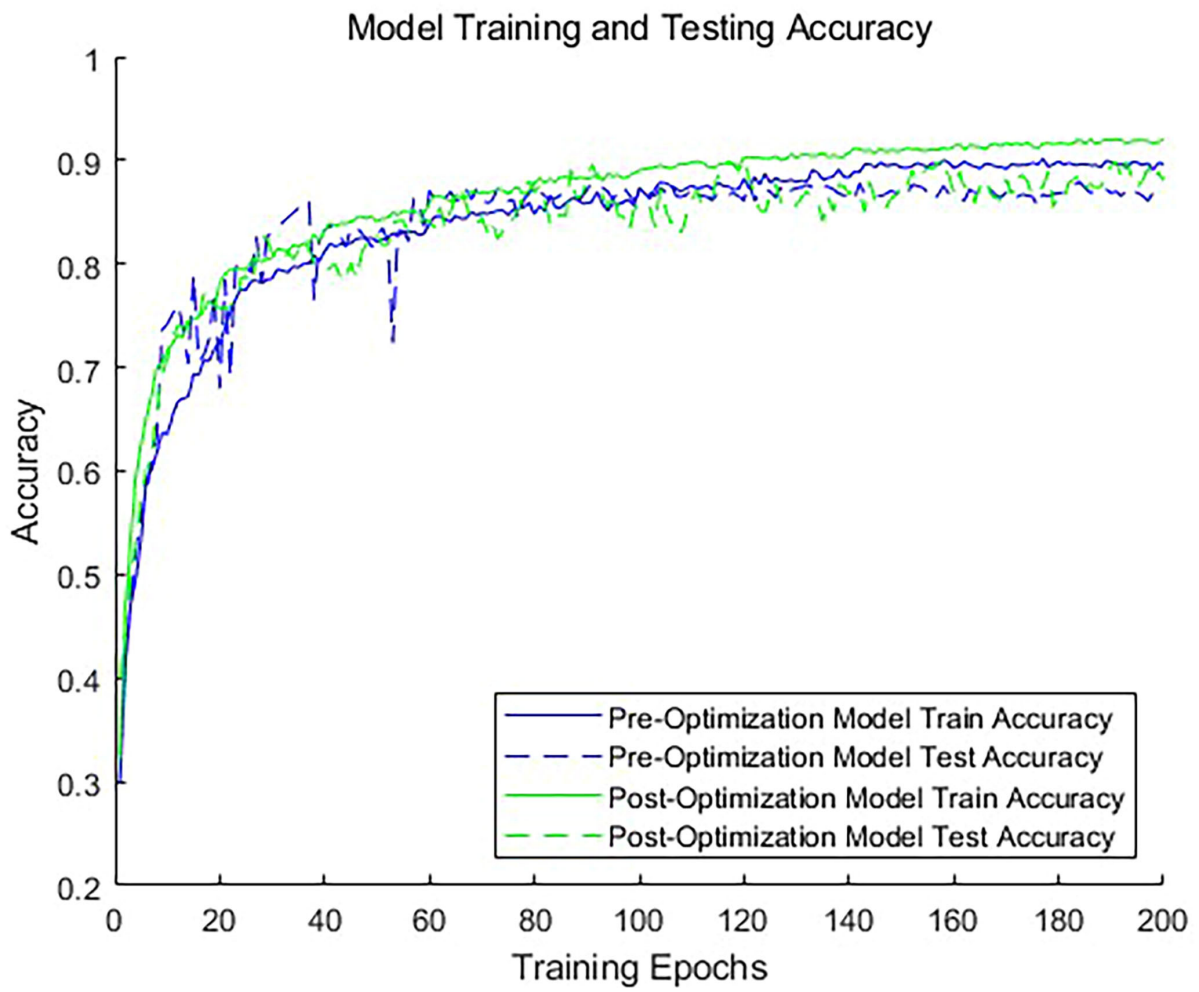
Accuracy of the Model Before and After Optimization.

To evaluate the impact of adding dimensionless features on the classification accuracy, we conducted comparative experiments. We trained two models: one using only a 1024-dimensional point cloud feature vector for classification, and the other incorporating a 1034-dimensional feature vector that includes dimensionless features. As illustrated in **Figure 8**, the inclusion of dimensionless features significantly improves the classification accuracy of the model. The model without dimensionless features achieved an accuracy of 89.6% for the validation set, whereas the model with dimensionless features achieved an accuracy of 92.4%. This demonstrates that incorporating custom dimensions can enhance the model accuracy. For the training duration, the optimized network required an average of 8 additional seconds per batch compared to the unoptimized network. It is clear that the inclusion of these 10 dimensionless features does not substantially reduce the operational efficiency of the network. This is due to the fact that these features are exclusively involved in the computations of the fully connected layers, representing only a small portion of the total 1034 features. Consequently, their impact on the overall computational burden is limited. Given the improved model accuracy, this marginal increase in training time is justified. This approach not only enhances the model’s predictive performance but also maintains satisfactory training efficiency.

**Figure 9:**
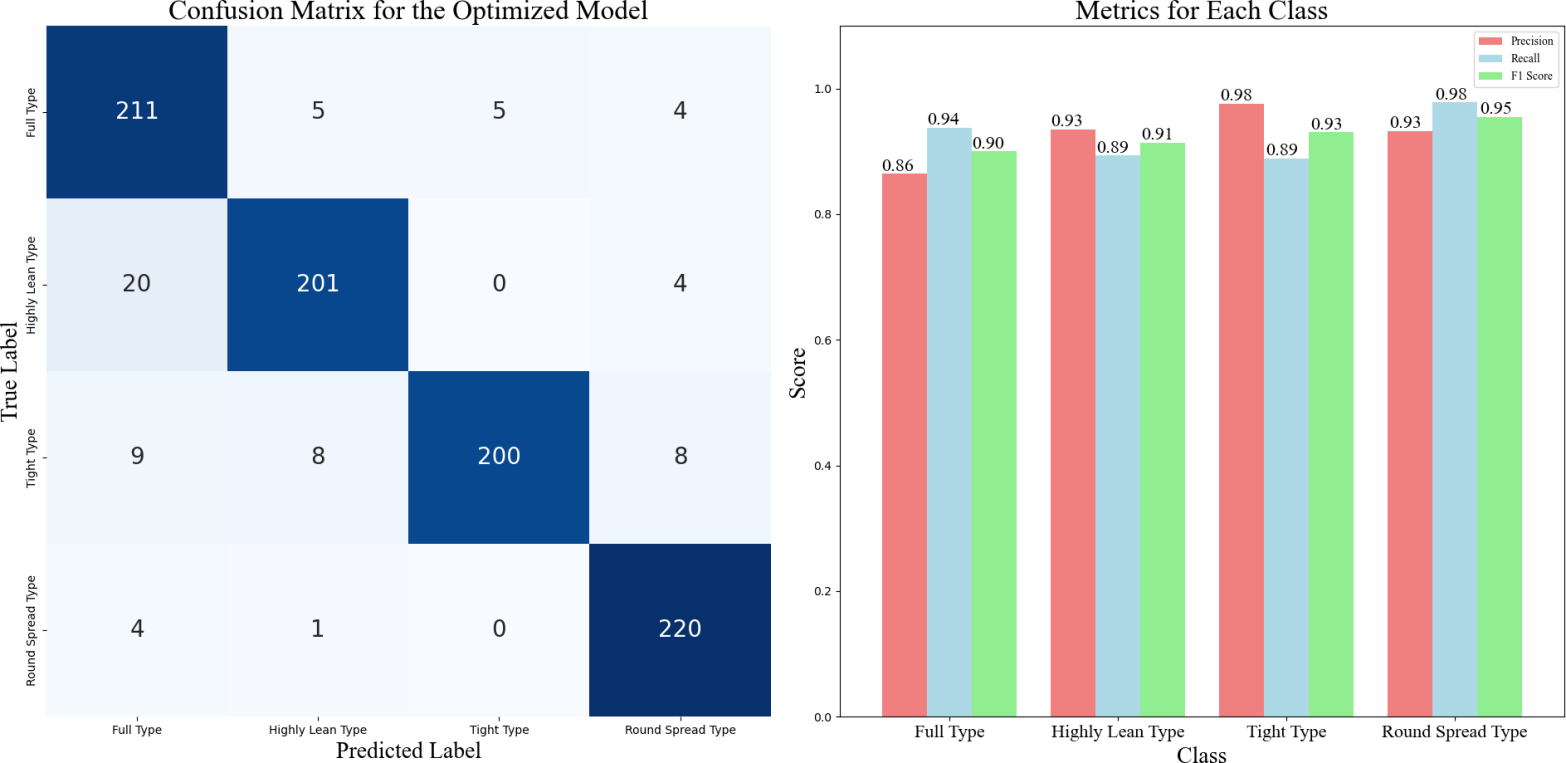
Confusion Matrix and Performance Metrics for the Optimized Model, noted: This figure shows how well the optimized model classifies the different types of Chinese cabbage plants. On the left is the confusion matrix, which tells us how often the model correctly or incorrectly predicts each of the four plant types. The numbers in the grid show the model’s predictions against the actual labels for each type. On the right, we see precision, recall, and F1-score for each type, which help us understand how well the model performs for each category.

The confusion matrix for the optimized model is shown in **Figure 9**. The confusion matrix summarizes the results of the classifier predictions, with all correct predictions located on the diagonal of the table. It is evident that the classification accuracy varies among different types of Chinese cabbage plant shapes. The round spread type had the highest classification accuracy, whereas the highly lean type had the lowest accuracy. This discrepancy may be due to the more distinct distinguishing features of the round spread type, which make it easier for the model to identify and classify them. In many cases, the high-lean type is misclassified as the full type because some characteristics of the high-lean type resemble those of the full type, leading to misclassification. This also suggests that the ten dimensionless parameters chosen, when most of them exhibit similar values, can result in classification errors.

To further validate the effectiveness of our method, we compared it with four advanced point cloud processing models: PointCNN, DGCNN, SPLATNet, and PointNet, using the same dataset. From **Table 3**, it is evident that our method outperforms these models in classification accuracy, average recall, and average F1 score. Our method achieved a classification accuracy of 92.4%, average recall of 92.5%, and average F1 score of 92.3%, all of which are higher than the respective metrics for PointCNN, DGCNN, SPLATNet, and PointNet. This demonstrates the leading performance of our approach in handling complex Chinese cabbage plant types.

**Table 3:** Comparative Analysis of Network Performance Metrics Across Different Models.

Model	Classification Accuracy (%)	Average Recall (%)	Average F1 Score (%)
Our Method	92.4	92.5	92.3
PointCNN	89.5	88.8	88.8
DGCNN	87.7	87.8	87.5
SPLATNet	90.1	90.0	90.0
Pointnet	79.1	79.2	77.5

## 4 Discussion

### 4.1 Method advantage and innovation

In previous studies, researchers often relied on two-dimensional images in order to classify distinct plant types. For example, Andono et al. (Andono et al., 2021). used Support Vector Machine (SVM), Naive Bayes, and K-Nearest Neighbor (KNN) algorithms to classify 15 types of orchids, training with 2250 images, and testing with 1500 images. The results showed that the SVM with a linear kernel and feature extraction had the highest accuracy of 98.13%, outperforming the Naive Bayes and KNN algorithms. Komi et al. (Komi et al., 2007) in their study proposed a novel weed detection and classification system that combined low-cost RGB and color spectral cameras. Under controlled laboratory lighting conditions, the system achieved 97.6% accuracy in identifying nonoverlapping whole leaves using samples from six different plant types. Although these methods are effective at distinguishing significantly different plant types, their limitations become apparent when classifying variations within the same plant species. Two-dimensional images fail to fully capture the three-dimensional structure and subtle features of plants, making them unsuitable for distinguishing plant shapes within the same species. In contrast, our method not only differentiates various plant species but also effectively addresses the classification of subtle shape differences within the same species.

Due to the limitations of two-dimensional data, in recent years, an increasing number of scholars have turned to three-dimensional data for plant classification research. Qian Y. et al. (Qian et al., 2021) used a Raytrix light field camera to obtain 3D point cloud data of rice seeds. After filtering, segmentation, and downsampling, the data were input into an improved PointNet network for feature extraction and classification. The improved PointNet model enhanced the rice variety classification accuracy to an average of 89.4% by adding cross-layer feature connections. Xi et al. (Xi et al., 2024) proposed a soil particle roundness classification method based on deep learning, achieving a classification accuracy of 92.19% on 2400 soil particle point cloud data points using the PointNet++ model, effectively handling defective particles. In comparison, the improved PointNet++ model achieved an accuracy of 92.4%, surpassing that of Qian et al. Our improvements in feature extraction and cross-layer feature fusion effectively capture subtle differences among categories, thereby enhancing the classification accuracy. Xu et al. (Xu et al., 2024) proposed the D-PointNet++ model, which achieved an overall classification accuracy (OA) of 92.65% and a mean class accuracy (mAcc) of 92.54% on the Nursery dataset. The model also obtained an average Intersection over Union (mIoU) of 89.90% and a mean class accuracy (mAcc) of 92.18% for segmentation tasks. These results demonstrate the significant advantages of the D-PointNet++ model in both tree species classification and tree part segmentation, further validating the effectiveness of the dense connection pattern and feature fusion operations in maintaining high classification accuracy even when processing highly complex point cloud datasets. Although our model’s accuracy is slightly lower than that of D-PointNet++, our research focuses on fine-grained classification of individual plant phenotypes within the same species, which is a more challenging task compared to the classification of different species. Despite this increased complexity, our model achieves comparable accuracy, highlighting its robustness and effectiveness in capturing subtle differences among plant phenotypes. This underscores the advanced capabilities of our model in handling intricate and nuanced data for plant phenotype classification.

Furthermore, in our species classification approach, we abandoned the traditional manual division and labeling method and innovatively adopted the quantified extraction of representative information combined with K-medoid clustering analysis. This method ensures more accurate dataset division. By objectively identifying and classifying the categories, we ensured the independence and scientific nature of the training data, thereby laying a solid foundation for further improving the accuracy of the classification model. This automated data preprocessing step reduces human error and enhances the generalization capability of the model across different datasets.

### 4.2 Limitations and future research directions

Despite these achievements, the study has some limitations. First, the diversity and scale of the dataset require further expansion to enhance the robustness and generalizability of the model. The current dataset primarily focuses on Chinese cabbage grown under the same environmental conditions and lacks broad coverage of other plant types or different environments, which may limit the model’s effectiveness across various settings and varieties; therefore, future research should aim to collect a wider range of image data from different plant species and growing conditions to better address these limitations. Specifically, measures can include: increasing plant types by collecting image data from various species, including common vegetables, fruits, and cereal crops, to validate the model’s applicability and effectiveness across a broader range of plant types; diversifying growing conditions by gathering plant images under different environmental conditions, such as greenhouses, fields, and different climate zones, to cover a variety of lighting, humidity, and soil conditions, thereby improving the model’s environmental adaptability; and expanding sample size by increasing the number of samples for each plant type to enhance the robustness of the model.

Second, more parameters will be extracted in the future. According to the feature characteristics of the misclassified results in this study, when most of the ten dimensionless parameters exhibit similar values, it can lead to classification errors. To improve distinction and accuracy, we can further increase the number of dimensionless parameters, such as Petiole length and thickness, leaf texture, and vein distribution, which may show greater differences between high-lean and full types, helping the model classify more accurately; additionally, using feature selection methods, such as recursive feature elimination and importance-based feature selection, can help identify the most relevant features and avoid introducing redundant parameters that may affect model efficiency.

Through continuous optimization and expansion, we expect this method to provide more scientific and efficient solutions for smart agricultural development and precise crop management.

## 5 Conclusion

In this study, 3D point-cloud data processing and the deep learning algorithm PointNet++ were used to quantify and classify Chinese cabbage plant shapes. By combining plant shape parameter extraction and the K-medoids clustering method, we achieved the precise quantification of Chinese cabbage plant shapes. We also optimized the basic PointNet++ algorithm by adding 10 plant shape features to the 1024 features automatically defined by PointNet++, improving the classification accuracy by 2.8% to 92.4%. Dimensionless features simplified the comparison between different categories, further revealing essential differences among various Chinese cabbage plant types. These features provide key information about cabbage morphology, creating more distinct classification boundaries in the feature space, and thus helping the model distinguish different categories more accurately. This approach not only provides a new method for the precise classification of agricultural crops but also serves as a reference for classification research in other fields.

This method provides a new approach for the precise classification of similar agricultural crops and demonstrates significant potential in smart agricultural systems. In terms of crop monitoring, the morphological features of plants can reflect their growth status and health conditions in real-time. This study offers farmers a unified method for managing plants, enabling them to promptly gather phenotypic information. In the area of yield prediction, the morphological features of plants are closely related to their final yield. Using our method, we can analyze the relationship between plant morphological features and yield, thereby predicting the final crop yield more accurately. This helps farmers develop more scientific planting plans, optimize field layouts, and select the most suitable growing areas for specific plants, leading to improved overall yield and economic efficiency. In disease management, the morphological features of plants change subtly when they are affected by diseases. Our method can capture these subtle changes, enabling early disease detection and providing a basis for timely treatment. Additionally, it not only detects whether plants are diseased but also classifies different disease types based on the differences in morphological features, helping farmers implement targeted control measures and reduce losses.

In summary, this study presents an effective method for the classification of Chinese cabbage plant shapes based on 3D point-cloud data, demonstrating excellent performance in highly accurate classification tasks and providing strong support for intelligent and precise management in the agricultural field.

## Data Availability

The raw data supporting the conclusions of this article will be made available by the authors, without undue reservation.
